# Motivations and expectations for using cannabis products to treat pain in humans and dogs: a mixed methods study

**DOI:** 10.1186/s42238-020-00045-x

**Published:** 2020-10-14

**Authors:** Jean E. Wallace, Lori R. Kogan, Eloise C. J. Carr, Peter W. Hellyer

**Affiliations:** 1 Department of Sociology, University of Calgary, 2500 University Drive NW, Calgary, AB T2N 1N4 Canada; 2 College of Veterinary Medicine and Biomedical Sciences, Colorado State University, Fort Collins, CO 80523 USA; 3 Faculty of Nursing, University of Calgary, 2500 University Dr NW, Calgary, AB T2N 1N4 Canada

**Keywords:** Medical cannabis, Chronic pain, Pain management, Well being, Canine, Alternative medicine

## Abstract

**Background:**

Social media and academic literature suggest that more people are using cannabis to treat their own or their dog’s chronic pain. This study identifies the reasons people use cannabis products to treat their own pain or their dog’s pain and explores whether these products have fulfilled their expectations.

**Methods:**

An anonymous, online survey was used to collect quantitative and qualitative self-report data on respondents’ perceptions, motivations and expectations about their or their dog’s chronic pain and cannabis use. The analyses are based on U.S. adults who reported using cannabis products to treat their own (*N* = 313) or their dog’s (*N* = 204) chronic pain. Quantitative responses from the two groups were compared using Chi-Square tests and qualitative data were analyzed using a thematic analysis.

**Results:**

Human patients and dog owners reported similar motivations for using cannabis products to treat chronic pain, with the more popular reasons being that cannabis products are natural, are preferred over conventional medication, are believed to be the best treatment or good treatment option for pain. Similar proportions of human patients and dog owners reported that the use of cannabis products fulfilled their expectations (86% vs. 82% respectively, χ^2^ (1, 200) = .59, *p* = .32). The qualitative data revealed that their expectations were met by reducing pain, increasing relaxation, and improving sleep, coping, functionality and overall well being. Additionally, the qualitative data suggests that cannabis products offer a return to normalcy and a restored sense of self to human and dog patients.

**Conclusions:**

The results suggest that people choose cannabis products because they are natural and a possible solution to managing chronic pain when conventional medicines have not been effective. Most people report that their expectations regarding pain management are fulfilled by these products. More accurate assessments are vital, however, for understanding both the objective biomedical and subjective socioemotional benefits of cannabis products for effective pain management for human and dog patients. In addition, objective factual information regarding cannabis products for effective pain management in humans and dogs is needed. It is recommended that both physicians and veterinarians work towards feeling more comfortable proactively broaching the subject of cannabis use with additional training and education.

## Background

Social media and academic literature suggest that more people are using alternative approaches to treat chronic pain. One alternative to conventional medicine that is receiving considerable attention is cannabis, especially in light of recent and rapid changes to U.S. legislation regarding the use and purchase of cannabis products. Currently, 33 U.S. states permit the use of marijuana for medical purposes and 11 additional states and the District of Columbia permit medical and recreational use (Wadsworth and Hammond 2020). The acceptance of cannabis products, for both recreational and medical purposes, among Americans continues to increase, with 91% of U.S. adults supporting the legalization of marijuana for medical use (Danieller 2019). Chronic pain relief is the most common use of medical marijuana, followed by arthritis and migraines (Park and Wu 2017). A review by Hill and his colleagues found that “pain relief is the most commonly cited reason for the medical use of cannabis” (2017:97).

Despite the increased clinical use of cannabis products, there remains considerable controversy regarding its efficacy and safety with only limited evidence to support its use under certain conditions (Hauser et al. 2017; Savage et al. 2016; Whiting et al. 2015). As more U.S. states legalize cannabis for medicinal purposes (and a projection of 11 more states are poised to pass legislation in 2020) (Marijuana Policy Project 2020), it is expected that increasing numbers of people will seek information and advice about the effectiveness of cannabis for pain management (Hill et al. 2017). Initial research results look promising. For example, a study recently published in *Journal of Psychoactive Drugs* (2019) found that 80% of people taking legalized marijuana reported it very or extremely helpful; leading to 82% of these people being able to reduce or stop taking over the counter pain medications, and 88% being able to stop taking opioid painkillers (Bachhuber et al. 2019). Another recent study supported this premise, noting that people using cannabis daily for pain management had a 50% lower odds of using illicit opioids compared to those not using cannabis (Lake et al. 2019).

In addition to treating human pain, many pet owners are considering cannabis products for their pets, especially as a therapeutic treatment for dogs suffering from chronic pain (Hartsel et al. 2019; Kogan et al. 2016; Kogan et al. 2019b; Kogan et al. 2020; Gamble et al. 2018). Pets are increasingly seen as members of the family and owners are willing to invest in their well-being; evidenced by an 6.1% increase from 2017 to 2018 in veterinary care spending (AVMA 2019). Yet due to the legal status of cannabis products, minimal research has been conducted on its potential therapeutic benefits for pets (Savage et al. 2016); instead research largely focuses on the harmful effects of marijuana ingestion by dogs (Brutlag, et al., 2018; McGrath et al. 2019). Due to the lack of research and legal restrictions, the FDA has not approved cannabis products for veterinary medical use and, in most states, veterinarians are legally prohibited from discussing medical cannabis treatment options. As a result, there is far less reliable evidence-based information available on the efficacy, dosage and safety of different delivery methods, appropriate doses and long term effects of cannabis products for dogs.

For these reasons, the majority of veterinarians are reluctant to talk about cannabis products with their clients even though many report observing positive signs in terms of chronic and acute pain, anxiety and seizure frequency or severity (Kogan et al. 2019a). Researchers are starting to address the pharmacokinetics of cannabis products in healthy dogs using different formulations and doses (see for example, Bartner et al. 2018, Deabold et al., 2019; Gamble et al. 2018; Kogan et al. 2020; Vaughn et al., 2020). These limited studies suggest that CBD-predominant oil formulations are safer and more tolerated in dogs than other methods (e.g., oral, transdermal creams (Bartner et al., 2018) or other formulations (e.g., THC-predominant oil, CBD/THC-predominant oil (Vaughn et al., 2020). A recent randomized placebo-controlled, veterinarian- and owner-blinded study reported that CBD-oil can significantly decrease pain and increase activity in dogs with osteoarthritis (Gamble et al. 2018). While the motivating factors, expectations and results regarding the use of cannabis products for oneself and one’s dog may be similar, there have been no studies that directly assess and compare these two populations.

In this study, we identify the most common reasons people are motivated to use cannabis products to treat their own pain or their dog’s pain. We also explore whether using cannabis to treat pain in themselves or their dog has fulfilled their expectations. The findings of this study shed light on the reasons people are increasingly turning to this alternative approach for pain management, as well as their beliefs and assumptions about the effectiveness of using these products for themselves or their dog. Knowledge about people’s expectations of the effectiveness of cannabis products can help health care providers and veterinarians direct conversations to better educate their patients and clients.

To address these questions, we carried out an embedded mixed methods study using two anonymous, online surveys in June 2019. These analyses are based on the responses of 313 people who reported using cannabis products to treat their own chronic pain and 204 pet owners who reported using cannabis products to treat their dog’s chronic pain.

## Data and methods

Two online, anonymous surveys were developed using Qualtrics (Qualtrics, Inc., Provo, UT, USA). One survey was designed for people who have used cannabis products for their own pain and the other survey targeted people who have used cannabis products for their dog’s pain. The surveys were similarly designed, with both surveys inquiring about motivations, expectations and experiences related to cannabis usage for pain. The surveys were designed, reviewed, and tested by the co-investigators and their colleagues. They were then pilot tested by five individuals for ambiguity and/or potentially missing or inappropriate response options, with revisions made based on the results. The final surveys and study design were approved by the Colorado State University Institutional Review Board (IRB # 19-9118H). Because the survey was anonymous, written informed consent was not required. Potential participants were provided with information about the study in the first page of the survey and instructed that by completing the survey they were providing informed consent. Confidentiality was ensured by the anonymity of the survey and because we did not collect any identifying information about individual participants or their dogs. Survey respondents received 50 cents for completing the survey, and this small amount suggests that respondents were internally motivated to complete the survey (Buhrmester et al. 2011).

Survey respondents were recruited through Amazon Mechanical Turk platform (MTurk; Amazon Inc., Seattle, WA, USA), an open online marketplace providing affordable access to over 100,000 potential survey respondents (Buhrmester et al. 2011). The diversity of participants recruited through MTurk is high (more diverse than typical Internet samples), and the quality of data collected meets the psychometric standards considered acceptable in published research (Buhrmester et al. 2011). Due to differences among countries regarding the legal status of cannabis products, the results are restricted to respondents residing in the United States.^1^ Adults (18 years or older) who either suffered from chronic pain or who currently owned at least one dog with chronic pain and had either used or thought about using cannabis for their own pain or their dog’s pain were the targeted audiences for the surveys. Chronic pain (human and canine) was defined for this study as pain that had lasted more than 3 months.

The surveys included closed-ended quantitative questions in combination with open-ended qualitative questions that invited participants to expand and clarify their thoughts and experiences. We asked participants about their attitudes and perceptions regarding their motivations, expectations and experiences with chronic pain and “cannabis products” for managing their pain. While some participants may use cannabis products for recreational purposes, our questions specifically asked them to share their opinions and experiences regarding “the use of cannabis products to treat their (or their dog’s) chronic pain”. It should be noted that establishing the accuracy of their pain diagnoses or use and clinical effectiveness of cannabis products are beyond the scope of this study. Our focus is on why people consider any form of cannabis product as a pain management option for themselves or their dog and whether they feel their expectations regarding these products have been met. The quantitative data are presented in the form of percentages indicating the frequency with which respondents selected certain responses.

While the purpose of this study is primarily descriptive, in some cases, we are able to see whether significant differences exist in the proportions of responses of human patients compared to dog owners. We use a chi-square test of homogeneity to test for differences in the proportions of categorical responses between the two groups. For items that allowed for multiple responses (i.e., types of products used and motivations for cannabis use), chi-square tests are not reported because they are inappropriate for items with categories that are not mutually exclusive.

The qualitative data were analyzed using a thematic analysis approach to identify and analyse patterns (Braun and Clarke 2006). Following familiarisation with the open text comments, codes were created to capture the semantic and conceptual understanding. Codes were then clustered into similar grouping to develop themes. This approach is well suited to a variety of different types of qualitative data and sizes of data sets, as well as analyses that are not theoretically driven (Clarke and Braun 2013).

### Measures

Respondents who were suffering from chronic pain were asked to indicate their **pain intensity** on the Numeric Rating Scale (Salaffi et al. 2004) that ranges from 0 (no pain) to 10 (worse pain ever). A numerical rating scale for pain measurement is seen as the gold standard for clinical pain research (Dworkin et al. 2005). Both human patients and dog owners were offered a list of **primary causes of chronic pain** informed by the World Health Organization’s International Classification (ICD) of Disease for chronic pain (Treede et al. 2015). Due to the significant disparity in the life spans of humans and dogs, different time frames were used to capture the **length of time** a person or dog had lived with chronic pain. Respondents were asked to indicate the **type of cannabis products used**. We offered respondents the following definitions of cannabis products derived from Hudock (2020):

- Marijuana/Cannabis - (THC > 0.3%)
- CBD/Hemp “Isolate” – (THC < 0.3%) --- CBD Isolate contains singular isolated CBD molecule only, no THC or other cannabis, terpenes, flavonoids, etc.
- CBD/Hemp - “Full Spectrum” or “Broad Spectrum” -- Full Spectrum refers to a raw, minimally processed extract that contains phytocannabis (i.e. CBD, CBD-A, THC, THC-A, etc.), terpenes, flavonoids, fatty acids, and other beneficial compounds. Broad Spectrum refers to a Full Spectrum extract that has been further processed to have the THC removed, as well as some of the other beneficial compounds.

The most frequent **way of obtaining cannabis products** was measured by a single item with the following responses: online sources, dispensary/store, natural/health store/service, given to me by friend/family, grown myself, and other. **Motivations for using cannabis to treat chronic pain** were determined by 23 reasons derived from Kogan’s 2016 and 2018 study (Kogan et al. 2016; Kogan et al. 2018). Respondents could select multiple reasons and they were also provided an open-ended “other” option. Respondents to asked to indicate whether the use of cannabis to treat their or their dog’s chronic pain has **fulfilled their expectations** by checking yes or no. They were then asked to explain their response in more detail in an open-ended question. These explanations are the qualitative data that were used in the thematic analysis described below.

### Description of the samples

All responses analyzed in this paper are based on people who have lived with chronic pain for more than 3 months (*N* = 313) or who have a dog with chronic pain for more than 3 months (*N* = 204). For respondents experiencing chronic pain themselves, their self-reported mean level of pain intensity was 6.67 on the Numeric Rating Scale that ranges from 0 (no pain) to 10 (worse pain ever).

Table 1 offers descriptive information on the causes and length of time living with chronic pain for respondents and their dogs. The two groups differ significantly in their primary sources of chronic pain (χ^2^ (3, *N* = 517) = 67.22, *p* < .001). About half of the respondents who suffer from chronic pain indicated it was due to chronic back pain (48%), whereas about half of the respondents with dogs with chronic pain indicated it was due to degenerative joint disease (45%). The majority of both groups had experienced chronic pain for over 1 year and many for much longer.

**Table 1:** Comparisons by Causes and Length of Time with Chronic Pain for Human Patients versus Dog Patients

Human Patients (***N*** = 313)		Dog Patients (***N*** = 204)	
**Primary Cause of Chronic Pain**		**Primary Cause of Chronic Pain**	
Chronic Back Pain	48%(149)	Chronic Back Pain	22% (45)
Degenerative Joint Disease	17% (54)	Degenerative Joint Disease	45% (91)
Mouth Pain or Headache	17% (52)	Mouth Pain from Dental Disease	7% (14)
Other	18% (58)	Other	26% (54)
χ2 (3, *N* = 571)=67.22, *p* < .001			
**Length of Time with Chronic Pain**		**Length of Time with Chronic Pain**	
< 1 year	18% (55)	< 1 year	36% (74)
1-5 years	47% (146)	1-3 years	55% (112)
> 5 years	35% (108)	> 3 years	9% (18)
χ2 (2, *N* = 513) = 52.26, *p* < .001			

Table 2 shows considerable variation in the types of cannabis products used to manage their own pain compared to those managing their dog’s pain). Most respondents report using marijuana/cannabis products to manage their own pain (76%), about half (49%) use hemp isolates and one third (36%) use CBD/hemp broad or full spectrum products. In contrast, the most commonly used product for managing their dog’s pain was hemp isolates (44%), followed by CBD/hemp broad or full spectrum products (42%). Marijuana/cannabis products were used by 26% of respondents to manage their dog’s pain.

**Table 2:** Descriptive Information for Cannabis Products Used and Comparison for How Cannabis Products are Obtained for Human Patients and Dog Patients

Human Patients (***N*** = 313)		Dog Patients (***N*** = 204)	
**Type of Cannabis Product Used**^a^		**Types of Cannabis Product Used**^a^	
Marijuana/Cannabis (THC > 0.3%)	76% (237)	Marijuana/Cannabis (THC > 0.3%)	26% (53)
Hemp Isolate (THC < 0.3%)	49% (152)	Hemp Isolate (THC < 0.3%)	44% (89)
CBD/Hemp Broad or Full Spectrum	36% (113)	CBD/Hemp Broad or Full Spectrum	42% (88)
Not Sure	3% (9)	Not Sure	11% (22)
**Most Frequent Way of Obtaining Cannabis**		**Most Frequent Way of Obtaining Cannabis**	
Given by Friend or Family	33% (102)	Given by Friend or Family	11% (22)
Dispensary or Store	30% (92)	Dispensary or Store	25% (50)
Natural/Health Store/Service	12% (40)	Natural/Health Store/Service	25% (50)
Online Source	12% (36)	Online Source	34% (68)
Other	13% (43)	Other	5% (14)
χ2 (4, *N* = 517) = 69.87, *p* < .001			

^a^Participants could select more than one type of cannabis product

In terms of where they most frequently obtain cannabis products, the two groups differ significantly (χ^2^ (4, *N* = 517) = 69.87, *p* < .001). Those obtaining cannabis products to manage their own pain rely primarily on friends or family (33%) or a dispensary or store (30%). In contrast, those obtaining it for their dog rely primarily on online sources (34%), or stores such as a dispensary (25%) or a natural or health store (25%).

## Motivations for using cannabis products to treat chronic pain

We asked participants to identify their motivations for using cannabis products to manage their pain or their dog’s pain from 23 different reasons listed in the survey. Respondents could select more than one reason from the list offered. It worth noting that of the 23 reasons offered, the most commonly selected motivations are the same six selected by both human patients and dog owners as shown in Table 3.

**Table 3:** Most Common Motivations for Using Cannabis Products to Treat Chronic Pain for Human Patients and Dog Patients

Motivations^a^	Human Patients (***N*** = 313) % (N)	Dog Patients (***N*** = 204) % (N)
I like the idea that this product comes from “natural” sources	55% (171)	43% (88)
I thought it was the best treatment for pain	50% (157)	56% (114)
I prefer cannabis/CBD products to conventional medicine	50% (156)	41% (83)
Because I thought it would a good treatment option	45% (140)	44% (90)
Other medications did not (do not) control the pain adequately	39% (121)	23% (48)
Recommendation from family or friends	37% (116)	30% (62)

^a^Participants could select more than one reason

Approximately one half of both groups selected the same four reasons: cannabis products are natural, preferred over conventional medication, seen to be the best treatment for pain, and a good treatment option. More of the respondents treating their own pain (39%) than their dogs’ pain (23%) reported feeling other medications do not control the pain adequately. Interestingly, approximately one-third of both groups indicated that one of the reasons they started using cannabis was because it was recommended by family or friends. While the list included the reason that it was recommended by their physician or veterinarian, only 9% (*N* = 27) of the human patients and 12% (*N* = 25) of the dog owners selected this reason.

In addition to the list of potential motivating factors, respondents were asked to share their reasons in their own words. From these open-ended qualitative responses, three main themes emerged that reflected the different reasons respondents use or have tried using cannabis products. These included the **availability** of the product, that it had been **recommended** to them by a friend (or occasionally by a health care professional), and that it **alleviated their symptoms**. A related but separate theme was ‘**it works best’**, which suggests that they had tried other products or interventions, but none had been as helpful.

## Expectations for using cannabis products to treat chronic pain

Table 4 shows that most respondents, whether they reported using cannabis products to manage their own pain or their dog’s pain, feel it met their expectations (own pain 86%; dog pain 82%). The two groups do not differ significantly in this regard (χ^2^ (1, *N* = 503) = 1.00, *p* = .317 (with Yates’ continuity correction). In addition, we examined the percentages of met expectations for human patients versus dog owners’ for each of the variables presented in Tables 5, 6 and 7 (see Appendix). The percentages of respondents who felt their expectations were met are comparable across all three tables for human patients and dog owners. That is, despite differences between the two groups shown in Tables 5, 6 and 7, none of these factors are differentially related to met expectations for human patients versus dog patients.

**Table 4:** Comparison by Met Expectations of Cannabis Products for Human Patients versus Dog Patients

Responses	Human Patients % (N)	Dog Patients % (N)
Yes	86% (261)	82% (162)
No	14% (44)	18% (36)
Total	100% (305)	100% (198)

χ^2^ (1, *N* = 503) = 1.00, *p* = .32 (with Yates’ continuity correction)

Respondents were asked why they feel their expectations were met, and analyses of their responses identified several core themes. **Reduced pain**, **greater relaxation**, **better sleep**, and **improved coping** were the main themes explaining how cannabis products met the expectations of people suffering from chronic pain. Similar themes were identified by dog owners that included **increased activity**, **reduced pain**, **greater relaxation, better sleep** and **improved well being**.

### Reduced pain

The majority of comments from people with chronic pain focused on their perception of experiencing reduced pain from using cannabis products. Many felt the effect on their pain was significant: *‘My daily pain has been reduced from a daily of 9 down to 2-4’*, ‘*Cannabis does substantially ease the pain’,* and ‘*It works so much better than the pain medicine it is just such a relief’.* Because dogs are non-verbal and cannot offer self reports of their pain, owner’s subjective observations of behavioral changes are the principal indicators to assess their dog’s pain (Goldberg 2017). Many dog owners referred to their dog’s increased activity and mobility when discussing their perceived pain reduction in their dog. For example ‘*She is now able to get up from sitting down without it being an ordeal for her – she is walking better and not so cranky so I can tell the CBD is totally working for her*’*, ‘He is a little stiff and sometimes is slower getting around. When we give him cannabis treats he runs and trots like he has no pain*’, and ‘*My dog is able to walk around more without being in obvious pain’*.

### Greater relaxation

The reduction of anxiety and relaxing effects were reported by numerous chronic pain sufferers: *‘I feel way more relaxed’ and ‘I have had great success with using cannabis and CBD cannabis strains to treat my pain. It helps relax me and forget about it, and puts my body at ease for a while’.* A reduction of anxiety was also observed by many dog owners. Dog owners referred to their dogs as being calmer, more relaxed, and less distressed: ‘*He’s able to be more relaxed and playful, before he was just lying around*’.

### Better sleep

The products were also felt to help people with chronic pain sleep better - both in terms of reducing their pain to facilitate sleep and also getting to and staying sleep: *‘It eases my pain and helps me sleep much better’* and *‘I use it so I can fall asleep and stay asleep at night’.* This was also mentioned by dog owners who felt that cannabis products helped their dog rest and sleep better: *‘The CBD products I have used have enabled my dog to rest better and be more active’, ‘She seems more relaxed and can sleep for longer periods of time’* and simply put *‘It helps Milo sleep better’.*

### Coping and well-being

Many respondents suffering from chronic pain who used cannabis products alluded to a general improvement in function and mood; thereby making life more manageable and easier to cope with: *‘Cannabis has significantly improved my ability to function day to day’*, *‘Cannabis really helps with the pain and makes life more manageable’*, and *‘It helps with the pain and makes me have a more positive outlook’.* Dog owners described the benefits of cannabis in terms of how they observed signs that they felt indicated their dog was happier, felt better and was in good spirits – all of which can be indicators of effective pain management (Epstein et al. 2015). For example, ‘*After using CBD she was a different dog and more herself full of energy and excitemen*t’ and ‘*My sweet girl Coco seems like her old self again, getting up and being active’.* Others described how their dog could *‘get back to being a fun-loving dog’* and how their dog is ‘*in higher spirits and energy level has resumed’*. Impressions from both human patients and dog owners suggest cannabis products led to an overall improvement in well-being in terms in mood, happiness and being better able to cope with and enjoy day-to-day activities.

### Did not meet their expectations

For those who felt that cannabis products did not meet their expectations in treating their chronic pain, the main theme was that they felt the product **not working** or helping in relieving their symptoms. Some respondents offered no specifics; just an overall comment ‘*Didn’t work’* or *‘Doesn’t feel like it works.* However, many commented about a lack of pain relief: *‘It doesn’t help my pain at all’, ‘I used CBD oil and it did nothing for my pain and made me too tired to function’.* For others, they felt that there was a reduction in their pain but it was not enough: ‘*It makes my pain tolerable, but it doesn’t take it away’.* Similarly, some dog owners simply reported the products did not appear to reduce signs of their dog’s pain while others mentioned the difficulty in determining whether it is benefitting their dog. Other owners felt it may have minimal, sporadic or temporary benefits in relieving their dog’s chronic pain. For example, ‘*It’s hard to see any relief’*, ‘*I cannot really tell if it works or not. I am afraid to increase the dose too much because I do not want to sedate her too much’, ‘CBD products I have used only relieve temporary conditions*’ and ‘*It seems to help sometimes but not always, and I guess I was hoping for total improvement*’. Comments from human patients and dog owners suggest that additional information about realistic expectations of pain relief, dosages and side effects of cannabis products may be helpful.

## Discussion

We found that people using cannabis products to manage their own pain differ in the products they use and how they obtain them in comparison to people using cannabis products to manage their dog’s pain. This may reflect variances in the laws and regulations regarding cannabis products, available forms of cannabis products for human and pet uses, and differences in their availability for purchase. Given that many respondents are obtaining cannabis products through informal and unregulated sources, it raises the question about whether they are adequately informed about proper usage, potential contraindications, dosages and risks. A troubling finding is the common reliance, for both groups, on informal and unregulated sources of information. These sources, namely friends or family and online sources, may not provide accurate information needed about the use, dosage, benefits, and side effects of these products. Previous research suggests that not only are internet health websites often misleading and inaccurate, people often do not investigate websites’ credibility (Kogan et al. 2014).

The quantitative results suggest that both groups have similar reasons for using cannabis products for pain management. Frequently cited motivations for choosing cannabis products are that they are natural, safe with no side-effects, and not controlled by pharmaceutical industries; and therefore preferred over conventional medication. Other studies have reported similar motivations for selecting cannabis products to by patients (e.g., Kruger and Kruger 2019) as well as pet owners (Kogan et al. 2016; Kogan et al. 2019a, 2019b). These studies also illustrate how participants emphasize that the natural qualities of cannabis products, which are viewed as safer and with fewer side effects compared to pharmaceutical drugs. As well, participants also report greater trust in medical cannabis products and providers than main stream healthcare or “big pharma” (Ryan and Sharts-Hopko 2017) .

The research on cannabis products for medicinal use, however, highlights the complexity and controversy regarding its efficacy and safety (Hauser et al. 2017; Savage et al. 2016; Whiting et al. 2015). Additionally, despite the perception of and preference for supporting small privately-owned businesses, cannabis companies are quickly becoming consolidated and acquired by large pharmaceutical companies. For example, 70% of both the USA and Canada’s top cannabis patent holders are major multinational pharmaceutical companies (Jones 2019).

The other popular reason for using cannabis products is that they are seen as a good, or even the best, treatment for pain, particularly when other medications do not control the pain. Our results are consistent with other qualitative studies that have found patients using medical cannabis products report greater relaxation, improved sleep and better day-to-day functioning with less side effects than conventional approaches (Lavie-Ajayi and Shvartzman 2019). As well, a recent meta-analysis of quantitative studies shows that pain, anxiety and mood/depression are among the most common reasons patients seek and use medical cannabis (Kosiba et al. 2019). Together, these results suggest that cannabis might be included as part of a multimodal approach to managing chronic pain that not only treats pain symptoms but also comorbid symptoms such as anxiety and depression. In reducing the negative emotional components associated with chronic pain, patients are more likely to adopt effective coping strategies that improve their overall well being (Ciaramella and Poli 2015). Similarly, recent studies on the use and benefits of cannabis products for dogs identified signs of pain relief, reduced inflammation, reduced anxiety and sleep aid as the main reasons for using these products (Kogan et al. 2016; Kogan et al. 2018).

As mentioned above, however, there is mixed evidence for the benefits of cannabis products for relieving human chronic pain. For example, a recent meta-analysis based on 91 publications representing 104 studies (*n* = 9958 participants) concluded “*It seems unlikely that cannabinoids are highly effective medicines for CNCP* [chronic noncancer pain]” (Stockings et al. 2018: 1951). Additionally, a recently published four-year prospective study of the effects of cannabis on chronic pain found that patients who used cannabis reported significantly *greater* pain severity than those not using cannabis (Campbell et al. 2018). In contrast, a study of 2987 users who completed 20,513 cannabis administration sessions over 2 years found that medical cannabis, especially when using higher THC products “*is associated with significant improvements in at least short-term pain relief*” (Li et al. 2019:128).

While rigorous research is lacking in regards to the use of cannabis products for canine pain relief, preliminary studies appear positive (Gamble et al. 2018; Kogan et al. 2020). Brutlag and Hommerding (2018) provide a comprehensive review of common types of cannabis products that cats and dogs may be exposed to, such as ingesting cannabis edible goods, plant material or prescription medicines, as well as recommendations for diagnosis and treatment plans for veterinarians. It has been suggested that dog owners might benefit from being educated on how to observe and interpret behavioral changes associated with pain as well as how to apply pain scoring tools (Epstein et al. 2015). This may not only offer more reliable pain assessments for pet owners and veterinarians but also may result in more effective pain management and care for their dog.

Our respondents also indicated how they felt their own sleep or their dog’s sleep had improved from using cannabis products to manage their chronic pain. Research on the benefits of cannabis products for sleep, and in particular, for those suffering from chronic pain, is still in its infancy (Babson et al. 2017). The studies that have been conducted are often based on small samples, and lack control variables and standard measures. As a result, the findings to date are mixed but do suggest cannabis products have the potential to help with sleep and pain (e.g., Piper et al. 2017), although long term, clinical trials are needed. There does not appear to be research on the relationship between dogs’ sleep quality and cannabis treatments.

Improvement in coping, functionality and quality of life were also identified as expectation areas fulfilled by using cannabis products to treat human and dog chronic pain. These findings are similar to those reported in Lavie-Ajayi and Shvartzman’s (2019) phenomenological study that involved interviews with chronic pain patients using medical cannabis. Their interviewees told researchers how the beneficial experiences of using cannabis go beyond the physiological effects. They described the benefits of cannabis as “a sigh of relief” and “a return to normality” – saying that it offers a sense of “restored self” from the overwhelming war against chronic pain that often takes over patients’ entire being and identity. A sense of normalcy and being able to cope with chronic pain may be related to feeling more in control over the situation. The authors note that restored self implies a sense of control over one’s life and regained sense of self that is highly subjective and not necessarily correlated with objective or biomedical markers.

Interestingly, we saw similar references and themes among the dog owners in which they referred to how their dog was a “new dog” or more like his or her “old self” before the pain started. This idea of a restored self for their dog was often expressed by comparing their dog’s activity levels or spirit before and after using cannabis products. Some owners talked about how their dog’s energy level, playfulness, or personality returned after using cannabis products to treat their pain. The comments from both human patients and dog owners highlight the subjective nature of the benefits of cannabis that may be difficult to detect in objective clinical measures, yet nevertheless, are critically important. This poses another methodological and clinical challenge in determining the benefits of cannabis use for medicinal purposes.

For those who did not feel cannabis products met their expectations, the overarching theme was it simply did not work; it failed to alleviate their pain. More detailed comments from human patients and dog owners indicated concerns about proper doses, temporary but not long-term relief, and sporadic but not total or continuous relief. Informed health care providers might be helpful in developing realistic expectations of the potential benefits of pain relief from cannabis products, as well as helping people make educated decisions about what to look for in a product and/or brand. As well, over the past several years, the U.S. Food and Drug Administration has tested the chemical content of new unapproved drugs that allegedly contain CBD and many were found that they do not contain the levels of CBD they claimed to contain (FDA 2019). This may offer a partial explanation of why consumers’ expectations are not being met, particularly if they are purchasing unapproved drugs. Consumers should be warned and educated about how to assess information regarding whether cannabis products are manufactured in compliance with FDA requirements, and whether they are assessed in an authorized laboratory and contain the amount of CDB that their label claims.

There are several limitations in the current study. The results are based on a small self-selected group of participants who were asked to *subjectively report* pain symptoms and cannabis effects Respondents’ reports did not include the extent to which symptoms objectively changed with cannabis use and we cannot verify respondents’ assessments of chronic pain in themselves or their dogs and changes resulting from the use of cannabis products. Moreover, while humans can articulate their perceptions of their own symptoms and perceived changes in them, dog owners must rely on observable behavioral signs in their dog’s behaviors, which may be highly subject to interpretation. Due to the uncertain legal status of cannabis products, detailed demographic information was not collected from participants or about their dogs to ensure complete anonymity in participating in the survey. As a result, we cannot assess the representativeness of our results but it is important to recognize that generalizability is not the goal of exploratory research such as this. As well, due to the variable nature of each brand and type of cannabis products, we did not inquire about specifics of cannabis products (e.g., type, dose) prescribed and/or used. As a result, we have limited information about the types and dosages of cannabis products that were used and how variations in types and dosages may relate to motivations and expectations. It is important to note, however, that the purpose of this study is not to demonstrate or promote the efficacy of cannabis products in managing chronic pain, but to explore subjective experiences and attitudes regarding motivations and expectations. Our findings suggest that most people are satisfied with the outcomes. More controlled clinical trials are needed to determine the extent to which different forms of cannabis products relate to positive outcomes for human and canine patients.

## Conclusions

The purpose of this paper was to identify reasons people are motivated to use cannabis products to treat their own pain or their dog’s pain and the extent to which the use of cannabis products to treat pain has fulfilled their expectations. We found that people choose cannabis products because they are natural and a possible solution to managing chronic pain when conventional medicines have not been effective. The use of cannabis products is believed to improve the quality of life for many humans and dogs suffering from chronic pain by reducing their pain, increasing relaxation, and improving sleep, coping, functionality, and overall well being. Challenges exist in assessing and evaluating these benefits objectively, whether for human or animal patients. More accurate assessments are vital for understanding both the objective biomedical and subjective socioemotional benefits of cannabis products for effective pain management.

Due to a lack of standardization of products as well as constant changes in legislation and regulation, it is difficult for patients, dog owners, health care providers, and veterinarians to acquire accurate information about the pain relief potential of cannabis products (Kogan et al. 2019a, 2019b; Mitchell et al. 2016). As a result, people are choosing cannabis products even though very few reported that their health care professional or veterinarian recommended them. We suggest that physicians and veterinarians acquire additional training and education so that they feel more comfortable proactively broaching the subject of cannabis use as a treatment option. The current laws and regulations give consumers minimal protection and information regarding cannabis products and companies, and many people obtain their information about cannabis products from friends, family or cannabis company websites. Objective data-driven websites, especially for cannabis products marketed for animals are rare (see FidoFortCollins.org for an example). By initiating the discussion of cannabis products, health care professionals give their patients and clients the message that they can be viewed as a resource for people seeking an alternative option to improve their own, and their dogs’, quality of life. Cannabis use for both people and animals is projected to grow, so future research that explores how to help all interested parties’ (e.g., patients, pet owners, health care professionals and veterinarians) feel more comfortable discussing cannabis products would be of benefit.

## Data Availability

The datasets used and/or analysed during the current study are available from the corresponding author on reasonable request.

## Appendix

**Table 5:** Percentages of Respondents Who Felt their Expectations were Met by Cause and Length of Time with Chronic Pain

Human Patients (***N*** = 313)		Dog Patients (***N*** = 204)	
**Primary Cause of Chronic Pain**		**Primary Cause of Chronic Pain**	
Chronic Back Pain	89% (129)	Chronic Back Pain	88% (38)
Degenerative Joint Disease	76% (41)	Degenerative Joint Disease	79% (70)
Mouth Pain or Headache	82% (40)	Mouth Pain from Dental Disease	79% (11)
Other	89% (51)	Other	79% (43)
**Length of Time with Chronic Pain**		**Length of Time with Chronic Pain**	
< 1 year	88% (45)	< 1 year	80% (57)
1-5 years	90% (128)	1-3 years	83% (91)
> 5 years	78% (83)	> 3 years	82% (14)

**Table 6:** Percentages of Respondents Who Felt their Expectations were Met by Cannabis Products Used and Obtained

Human Patients (***N*** = 313)		Dog Patients (***N*** = 204)	
**Type of Cannabis Product Used**^a^		**Types of Cannabis Product Used**^a^	
Marijuana/Cannabis (THC > 0.3%)	88% (199)	Marijuana/Cannabis (THC > 0.3%)	94% (45)
Hemp Isolate (THC < 0.3%)	83% (120)	Hemp Isolate (THC < 0.3%)	76% (68)
CBD/Hemp Broad or Full Spectrum	90% (99)	CBD/Hemp Broad or Full Spectrum	87% (75)
Not Sure	67% (6)	Not Sure	68% (15)
**Most Frequent Way of Obtaining Cannabis**		**Most Frequent Way of Obtaining Cannabis**	
Given by Friend or Family	86% (88)	Given by Friend or Family	77% (17)
Dispensary or Store	85% (78)	Dispensary or Store	84% (42)
Natural/Health Store/Service	90% (36)	Natural/Health Store/Service	84% (42)
Online Source	78% (36)	Online Source	82% (56)
Other	80% (20)	Other	63% (9)

^a^Participants could select more than one type of cannabis product

**Table 7:** Percentages of Respondents Who Felt their Expectations were Met and Motivations for using Cannabis Products

Common Motivations	Human Patients (***N*** = 313) % (N)	Dog Patients (***N*** = 204) % (N)
I like the idea that this product comes from “natural” sources	88% (149)	86% (76)
I thought it was the best treatment for pain	90% (142)	89% (101)
I prefer cannabis/CBD products to conventional medicine	92% (144)	99% (82)
Because I thought it would a good treatment option	79% (110)	81% (73)
Other medications did not (do not) control the pain adequately	85% (103)	85% (41)
Recommendation from family or friends	83% (96)	79% (49)

## Abbreviations

AVMA: American Veterinary Medical Association
CBD: Cannabidiol
CBD-A: Cannabidolic Acid
CNCP: Chronic noncancer pain
FDA: Food and Drug Administration
IRB: Institutional Review Board
MTurk: Mechanical Turk
THC: Tetrahydrocannabinol
THC-A: Tetrahydrocannabonolic Acid
U.S.: United States
USA: United States of America
WA: Washington

## Footnotes

1 Note that initially the survey was offered to people residing in both Canada and the United States. Due to the extremely small number of responses from Canadians using cannabis products (i.e., 12 from chronic pain sufferers and 8 from people with dogs with chronic pain), they have been excluded from the analysis.

## References

[CR1] AVMA (2019). Pet ownership, spending going strong. Accessed at: https://www.avma.org/javma-news/2019-06-01/pet-ownership-spending-going-strong. Accessed 7 Jan 2020.

[CR2] Babson KA, Sottile J, Morabito D (2017). Cannabis, cannabinoids, and sleep: a review of the literature. Curr Psychiatry Rep.

[CR3] Bachhuber M, Arnsten JH, Wurm G (2019). Use of cannabis to relieve pain and promote sleep by customers at an adult use dispensary. J Psychoactive Drugs.

[CR4] Bartner LM, McGrath S, Rao S, Hyatt LK, Wittenburg LA (2018). Pharmacokinetic of cannabidiol administered by 3 delivery methods at 2 different dosages to healthy dogs. Can J Vet Res.

[CR5] Braun V, Clarke V (2006). Using thematic analysis in psychology. Qual Res Psychol.

[CR6] Brutlag A, Hommerding H (2018). Toxicology of Marijuana, Synthetic Cannabinoids, and Cannabidiol in Dogs and Cats. Vet Clin Small Anim.

[CR7] Buhrmester M, Kwang T, Gosling S (2011). Amazon’s mechanical Turk: a new source of inexpensive yet high-quality, data?. Perspect Psychol Sci.

[CR8] Campbell G, Hall WD, Peacock A (2018). Effect of cannabis use in people with chronic non-cancer pain prescribed opioids: findings from a 4-year prospective cohort study. Lancet Public Health.

[CR9] Ciaramella A, Poli P (2015). Chronic low back pain: perception and coping with pain in the presence of psychiatric comorbidity. J Nerv Ment Dis.

[CR10] Clarke V, Braun V (2013). Teaching thematic analysis: overcoming challenges and developing strategies for effective learning. Psychologist.

[CR11] Danieller A. Two-thirds of Americans support marijuana legalization: Pew Research Center; 2019. Accessed at http://pewrsr.ch/2E9u3hd on 3 Feb 2020.

[CR12] Deabold KA, Schwark WS, Wolf L, Wakshlag JJ. Single-dose pharmacokinetics and preliminary safety assessment with use of CBD-rich hemp nutraceutical in healthy dogs and cats. Animals 2019;9:832. 10.3390/ani9100832. 10.3390/ani9100832 PMC6826847 31635105

[CR13] Dworkin RH, Turk DC, Farrar JT (2005). Core outcome measures for chronic pain clinical trials: IMMPACT recommendations. PAIN.

[CR14] Epstein ME, Rodan I, Griffenhagen G (2015). AAHA/AAFP pain management guidelines for dogs and cats. J Feline Med Surg.

[CR15] FDA (2019). Warning letters and test results for cannabidiol-related products.

[CR16] Gamble L-J, Boesch JM, Frye CW, Schwark WS, Mann S, Wolfe L, Brown H, Berthelsen ES, Wakshlag JJ (2018). Pharmacokinetics, Safety, and Clinical Efficacy of Cannabidiol Treatment in Osteoarthritic Dogs. Front Vet Sci.

[CR17] Goldberg ME (2017). A look at chronic pain in dogs. Vet Nurs J.

[CR18] Hartsel JA, Boyar K, Pham A, Silver, Gupta R, Srivastava A, Lall R (2019). Cannabis in veterinary medicine: Cannabinoid therapies for animals. Nutraceuticals in Veterinary Medicine.

[CR19] Hauser W, Petzke F, Fitzcharles MA. Efficacy, tolerability and safety of cannabis-based medicines for chronic pain management – An overview of systematic reviews. Eur J Pain. 2017;22(3). 10.1002/ejp.1118. 10.1002/ejp.1118 29034533

[CR20] Hill KP, Palastro MD, Johnson B, Ditre JW (2017). Cannabis and pain: a clinical review. Cannabis Cannabinoid Res.

[CR21] Hudock C (2020). Full Spectrum, Broad Spectrum, and Isolate: Meanings and Differences.

[CR22] Jones K (2019). The big pharma takeover of medicinal cannabis.

[CR23] Kogan L, Schoenfeld-Tacher R, Hellyer P, Rishniw M (2019). US veterinarians’ knowledge, experience, and perception regarding the use of cannabis for canine medical conditions. Front Vet Sci.

[CR24] Kogan LR, Hellyer PW, Downing R (2020). The use of Cannabidiol-rich hemp oil extract to treat canine osteoarthritis-related pain: a pilot study. AHVMA J.

[CR25] Kogan LR, Hellyer PW, Robinson NG (2016). Consumers’ perceptions of hemp products for animals. J Am Holist Vet Med Assoc.

[CR26] Kogan LR, Hellyer PW, Schoenfeld-Tacher R (2018). Dog owners’ use and perceptions of cannabis products. J Am Holist Vet Med Assoc.

[CR27] Kogan LR, Hellyer PW, Silcox S, Schoenfeld-Tacher R (2019). Canadian dog owners’ use of perceptions of cannabis products. Can Vet J.

[CR28] Kogan LR, Schoenfeld-Tacher R, Gould L, Hellyer PW, Dowers K (2014). Information prescriptions: a tool for veterinary practices. Open Vet J.

[CR29] Kosiba JD, Maisto SA, Ditre JW (2019). Patient-reported use of medical cannabis for pain, anxiety, and depression symptoms: systematic review and meta-analysis. Soc Sci Med.

[CR30] Kruger DJ, Kruger JS (2019). Medical cannabis users’ comparisons between medical cannabis and mainstream medicine. J Psychoactive Drugs.

[CR31] Lake S, Walsh Z, Kerr T (2019). Frequency of cannabis and illicit opioid use among people who use drugs and report chronic pain: a longitudinal analysis. PLoS Med.

[CR32] Lavie-Ajayi M, Shvartzman P (2019). Restored self: a phenomenological study of pain relief by cannabis. Pain Med.

[CR33] Li X, Vigil JM, Stith SS (2019). The effectiveness of self-directed medical cannabis treatment for pain. Comp Therapies Med.

[CR34] Marijuana Policy Project (2020). Marijuana Policy reform legislation.

[CR35] McGrath S, Bartner LR, Rao S, Packer RA, Gustafson DL (2019). Randomized blinded controlled clinical trial to assess the effect of oral cannabidiol administration in addition to conventional antiepileptic treatment on seizure frequency in dogs with intractable idiopathic epilepsy. J Am Vet Med Assoc.

[CR36] Mitchell F, Gould O, LeBlanc M, Manuel L (2016). Opinions of hospital pharmacists in Canada regarding marijuana for medical purposes. Can J Hosp Pharm.

[CR37] Park JY, Wu L-T (2017). Prevalence, reasons, perceived effects, and correlates of medical marijuana use: a review. Drug Alcohol Depend.

[CR38] Piper BJ, Beals ML, Abess AT (2017). Chronic pain patients' perspectives of medical cannabis. Pain..

[CR39] Ryan J, Sharts-Hopko N (2017). The experiences of medical marijuana patients: a scoping review of the qualitative literature. J Neurosci Nurs.

[CR40] Salaffi F, Stancati A, Silvestri CA, Ciapetti A, Grassi W (2004). Minimal clinically important changes in chronic musculoskeletal pain intensity measured on a numerical rating scale. Eur J Pain.

[CR41] Savage SR, Romero-Sandoval A, Schatman M (2016). Cannabis in pain treatment: clinical and research considerations. J Pain.

[CR42] Stockings E, Campbell G, Hall WD (2018). Cannabis and cannabinoids for the treatment of people with chronic noncancer pain conditions: a systematic review and meta-analysis of controlled and observational studies. PAIN.

[CR43] Treede RD, Rief W, Barke A, Aziz Q, Bennett MI, Benoliel R (2015). A classification of chronic pain for ICD-11. Pain.

[CR44] Vaughn D, Kulpa J, Paulionis L (2020). Preliminary investigation of the safety of escalating cannabinoid doses in healthy dogs. Front Vet Sci.

[CR45] Wadsworth E, Hammond D. Out-of-state cannabis purchases in the United States. Drug Alcohol Depend. 2020;207. 10.1016/j.drugalcdep.2019.107822. 10.1016/j.drugalcdep.2019.107822 31911336

[CR46] Whiting PF, Wolff RF, Deshpande S (2015). Cannabinoids for medical use: a systematic review and meta-analysis. JAMA.

