# Publisher Correction to: Motivations and expectations for using cannabis products to treat pain in humans and dogs: a mixed methods study

**DOI:** 10.1186/s42238-020-00048-8

**Published:** 2020-11-20

**Authors:** Jean E. Wallace, Lori R. Kogan, Eloise C. J. Carr, Peter W. Hellyer

**Affiliations:** 1 Department of Sociology, University of Calgary, 2500 University Drive NW, Calgary, AB T2N 1 N4 Canada; 2 College of Veterinary Medicine and Biomedical Sciences, Colorado State University, Fort Collins, CO 80523 USA; 3 Faculty of Nursing, University of Calgary, 2500 University Dr NW, Calgary, AB T2N 1 N4 Canada

**Publisher Correction to: J Cannabis Res 2, 36 (2020)**

**https://doi.org/10.1186/s42238-020-00045-x**

Following publication of the original article (Wallace et al. 2020), the authors reported that the article had published with errors in some of the tables; data had been erroneously omitted from Tables 1, 2 and 6, and these tables had been incorrectly formatted.

**Table 1:** Comparisons by Causes and Length of Time with Chronic Pain for Human Patients versus Dog Patients

Human Patients (***N*** = 313)		Dog Patients (***N*** = 204)	
**Primary Cause of Chronic Pain**		**Primary Cause of Chronic Pain**	
Chronic Back Pain	48%(149)	Chronic Back Pain	22% (45)
Degenerative Joint Disease	17% (54)	Degenerative Joint Disease	45% (91)
Mouth Pain or Headache	17% (52)	Mouth Pain from Dental Disease	7% (14)
Other	18% (58)	Other	26% (54)
χ2 (3, *N* = 571) = 67.22, *p* < .001			
**Length of Time with Chronic Pain**		**Length of Time with Chronic Pain**	
< 1 year	18% (55)	< 1 year	36% (74)
1-5 years	47% (146)	1-3 years	55% (112)
> 5 years	35% (108)	> 3 years	9% (18)
χ2 (2, *N* = 513) = 52.26, *p* < .001

**Table 2:** Descriptive Information for Cannabis Products Used and Comparison for How Cannabis Products are Obtained for Human Patients and Dog Patients

Human Patients (***N*** =313)		Dog Patients (***N*** = 204)	
**Type of Cannabis Product Used** ^a^		**Types of Cannabis Product Used** ^a^	
Marijuana/Cannabis (THC > 0.3%)	76% (237)	Marijuana/Cannabis (THC > 0.3%)	26% (53)
Hemp Isolate (THC < 0.3%)	49% (152)	Hemp Isolate (THC < 0.3%)	44% (89)
CBD/Hemp Broad or Full Spectrum	36% (113)	CBD/Hemp Broad or Full Spectrum	42% (88)
Not Sure	3% (9)	Not Sure	11% (22)
**Most Frequent Way of Obtaining Cannabis**		**Most Frequent Way of Obtaining Cannabis**	
Given by Friend or Family	33% (102)	Given by Friend or Family	11% (22)
Dispensary or Store	30% (92)	Dispensary or Store	25% (50)
Natural/Health Store/Service	12% (40)	Natural/Health Store/Service	25% (50)
Online Source	12% (36)	Online Source	34% (68)
Other	13% (43)	Other	5% (14)
χ2 (4, *N* = 517) = 69.87, *p* < .001			

^a^Participants could select more than one type of cannabis product

**Table 6:** Percentages of Respondents Who Felt their Expectations were Met by Cannabis Products Used and Obtained

Human Patients (***N*** = 313)		Dog Patients (***N*** = 204)	
**Type of Cannabis Product Used** ^a^		**Types of Cannabis Product Used** ^a^	
Marijuana/Cannabis (THC > 0.3%)	88% (199)	Marijuana/Cannabis (THC > 0.3%)	94% (45)
Hemp Isolate (THC < 0.3%)	83% (120)	Hemp Isolate (THC < 0.3%)	76% (68)
CBD/Hemp Broad or Full Spectrum	90% (99)	CBD/Hemp Broad or Full Spectrum	87% (75)
Not Sure	67% (6)	Not Sure	68% (15)
**Most Frequent Way of Obtaining Cannabis**		**Most Frequent Way of Obtaining Cannabis**	
Given by Friend or Family	86% (88)	Given by Friend or Family	77% (17)
Dispensary or Store	85% (78)	Dispensary or Store	84% (42)
Natural/Health Store/Service	90% (36)	Natural/Health Store/Service	84% (42)
Online Source	78% (36)	Online Source	82% (56)
Other	80% (20)	Other	63% (9)

^a^Participants could select more than one type of cannabis product

The original article has since been updated to correct the tables.

Furthermore, please find the (corrected) tables in this correction for reference.

The publisher apologizes for this error and any inconvenience caused.
